# Neighbourhood-level social capital, marginalisation, and the incidence of schizophrenia and schizoaffective disorder in Toronto, Canada: a retrospective population-based cohort study

**DOI:** 10.1017/S003329172100458X

**Published:** 2023-04

**Authors:** Martin Rotenberg, Andrew Tuck, Kelly K. Anderson, Kwame McKenzie

**Affiliations:** 1 Department of Psychiatry, University of Toronto, Toronto, ON, Canada; 2 Centre for Addiction and Mental Health, Toronto, Canada; 3 Department of Epidemiology & Biostatistics, Department of Psychiatry, The University of Western Ontario, London, ON, Canada

**Keywords:** Epidemiology, incidence, marginalisation, schizophrenia, social capital, socioenvironmental

## Abstract

**Background:**

Studies have shown mixed results regarding social capital and the risk of developing a psychotic disorder, and this has yet to be studied in North America. We sought to examine the relationship between neighbourhood-level marginalisation, social capital, and the incidence of schizophrenia and schizoaffective disorder in Toronto, Canada.

**Methods:**

We used a retrospective population-based cohort to identify incident cases of schizophrenia and schizoaffective disorder over a 10 year period and accounted for neighbourhood-level marginalisation and a proxy indicator of neighbourhood social capital. Mixed Poisson regression models were used to estimate adjusted incidence rate ratios (aIRRs).

**Results:**

In the cohort (*n* = 649 020) we identified 4841 incident cases of schizophrenia and schizoaffective disorder. A 27% variation in incidence was observed between neighbourhoods. All marginalisation dimensions, other than ethnic concentration, were associated with incidence. Compared to areas with low social capital, areas with intermediate social capital in the second [aIRR = 1.17, 95% confidence interval (CI) 1.03–1.33] and third (aIRR = 1.23, 95% CI 1.08–1.40) quintiles had elevated incidence rates after accounting for marginalisation. There was a higher risk associated with the intermediate levels of social capital (aIRR = 1.18, 95% CI 1.00–1.39) when analysed in only the females in the cohort, but the CI includes the possibility of a null effect.

**Conclusions:**

The risk of developing schizophrenia and schizoaffective disorder in Toronto varies by neighbourhood and is associated with socioenvironmental exposures. Social capital was not linearly associated with risk, and risk differs by sex and social capital quintile. Future research should examine these relationships with different forms of social capital and examine how known individual-level risk factors impact these findings.

Variation in the incidence of schizophrenia and other psychotic disorders is well described in the literature, with a consistent finding of a higher incidence in urban environments (Heinz, Deserno, & Reininghaus, 2013; March et al., 2008; Vassos, Pedersen, Murray, Collier, & Lewis, 2012) and more socially deprived areas (O'Donoghue, Roche, & Lane, 2016b). These findings have predominantly been replicated in studies conducted in Northern Europe and other high-income countries (Fett, Lemmers-Jansen, & Krabbendam, 2019). It has been proposed that urbanicity may be a proxy for other socioenvironmental risk factors which may include known risk factors such as social and economic marginalisation (which are ways people and groups are excluded from society), isolation, immigration, ethnicity, substance use, and other exposures which are each associated with an increased risk of developing a psychotic disorder to varying degrees (McGrath & Scott, 2006; Murray, David, & Ajnakina, 2020).

Social capital is one factor that has been studied as a potential factor that may play a role in the risk of developing a psychotic illness. As a concept, social capital broadly attempts to describe features of society that enable people to pursue shared objectives (McKenzie, Whitley, & Weich, 2002). Although it can be measured, defined, and conceptualised in numerous ways, it can generally be understood from social cohesion and social network approaches (Rotenberg, Anderson, & McKenzie, 2020). The social cohesion perspective focuses on community relationships (Ehsan, Klaas, Bastianen, & Spini, 2019) and can encompass a broad range of dimensions which include: (1) community networks, (2) civic engagement, (3) trust in the community, (4) local civic identity, and (5) reciprocity and norms of cooperation (Putnam, 2000). The network approach focuses on the resources available to people and communities through social networks (Villalonga-Olives & Kawachi, 2015).

As a construct, social capital may be important in the study of social risk factors for psychotic disorders, and may contribute to risk associated with other socioenvironmental exposures including social and material marginalisation and social isolation which may occur in urban environments (Rotenberg et al., 2020). It may also play a role as a driving factor behind the ethnic density effect, whereby a lower risk of psychosis has been found in ethnic minority persons who live in communities where there are more individuals who are of a similar background, potentially due to increased resources and support available (Morgan, Knowles, & Hutchinson, 2019). However, there remains only limited empirical evidence on the role of social capital, and its association with other social factors, in the aetiology of psychosis.

Of the few studies that have examined the relationship between social capital and the risk of psychosis, the results have been mixed (Rotenberg et al., 2020). Some studies have found low levels of social capital to be associated with a higher incidence of psychosis, whereas others have found no association. A non-linear relationship between social capital and psychotic disorders has also been described, with higher rates in neighbourhoods with both higher and lower levels of social capital, and lower rates of psychosis in areas with intermediate levels of social capital (Kirkbride et al., 2008). Further research has found the association between social capital and the risk of psychosis to be specific to particular demographic groups, including females (O'Donoghue et al., 2016a) and people with a family history of mental illness (Binbay et al., 2012).

The purpose of this study was to examine the relationship between indicators of social capital and marginalisation and the incidence of psychotic disorders, specifically schizophrenia or schizoaffective disorder, in Toronto, Canada, with a focus on variation at the neighbourhood level. The association between social capital and risk of developing a psychotic disorder has yet to be studied in North America. Moreover, Toronto is one of the most multicultural cities in the world, with a demographic profile that is different from other cities where these relationships have previously been studied. The current study uses health administrative data to determine the incidence of psychotic disorders in neighbourhoods across Toronto, and examine the relationship with neighbourhood-level indicators of marginalisation and social capital. We hypothesised that (i) neighbourhood marginalisation will be associated with the risk of developing a psychotic disorder, (ii) higher levels of neighbourhood social capital will be associated with a lower incidence of psychotic disorders, (iii) adjustment for social capital will attenuate the effects of neighbourhood-level marginalisation, and (iv) these associations will differ between males and females.

## Methods

### Study design, setting, and population

We used a subset of a population-based retrospective cohort which was initially constructed to estimate the incidence of schizophrenia and schizoaffective disorder among the entire population of the province of Ontario, and included all residents in the province between the ages of 14 and 40 as of 1 April 1999 (Anderson, Cheng, Susser, McKenzie, & Kurdyak, 2015; Rotenberg, Tuck, Anderson, & McKenzie, 2021). Briefly, the cohort was constructed using health administrative data held at ICES (formerly the Institute for Clinical Evaluative Science), which enables data linkages at the individual level.

People included in this cohort were followed for 10 years and were eligible for the single-payer Ontario Health Insurance Plan (OHIP) in the 5 years prior to cohort inception. To ensure an incident cohort, all people who had any previous contact with the provincial health system for a psychotic disorder up to 10 years prior to the cohort inception were removed from the sample (prevalent cases). The analytic sample for the current study was restricted to people from this cohort who resided in one of the 140 Toronto neighbourhoods at the time of cohort entry.

### Data sources

The following ICES data holdings were used: the Registered Persons Database (RPDB), which is a central population registry that contains basic demographic data for all Ontario residents insured by OHIP; the Ontario Mental Health Reporting System (OMHRS), which contains data on all inpatient hospitalisations to adult mental health beds; the Canadian Institute for Health Information Discharge Abstract Database (CIHI-DAD), which contains data on all acute care hospitalisations and inpatient psychiatric hospitalisations before 2005; the National Ambulatory Care Reporting System (NACRS), which contains data on emergency department visits; and outpatient physician billings from OHIP.

These data were linked to data from the Urban Health Equity Assessment and Response Tool (Urban HEART) Toronto (Centre for Research in Inner City Health, 2014), which provides indicators of neighbourhood-level health equity. These data were also linked to the Ontario Marginalisation Index (ON-Marg), which is an area-level deprivation index based on census data (Matheson, Dunn, Smith, Moineddin, & Glazier, 2012). Both linkages were based on six-digit residential postal code at the time of cohort entry.

### Incident cases

Incident cases of schizophrenia and schizoaffective disorder were identified over a 10 year follow-up period (1999–2008). Classification of incident psychotic disorder was based on: (i) a primary discharge diagnosis from an inpatient hospitalisation with a diagnosis of schizophrenia or schizoaffective disorder based on ICD-9 code 295.*x*, ICD-10 code F20 or F25, or DSM-IV code 295.*x*; or (ii) a minimum of two OHIP billing claims or emergency department visits with a diagnostic code for schizophrenia or schizoaffective disorder (ICD-9 code 295.*x*, or ICD-10 code F20 or F25) in a 12-month period. This method of case ascertainment has been validated against medical chart diagnoses, and found high sensitivity (91.6%), and moderate specificity (67.4%) (Kurdyak, Lin, Green, & Vigod, 2015). Person-time follow-up was calculated for each person in the cohort, from the time of the inception of the cohort until an index episode of a psychotic disorder, death, or end of the follow-up period.

### Covariates and exposure classification

Demographic variables – including age, sex, and place of residence – were defined at the time of cohort inception. Marginalisation and social capital were defined at the neighbourhood-level at the time of cohort entry via linkage of postal codes to one of the 140 Toronto neighbourhood geographies. The 140 Toronto neighbourhoods are based on census tract boundaries and are comprised of two to five census tracts per neighbourhood with a minimum population of 7000–10 000 people. These neighbourhood boundaries are used by government and community organisations in the planning of services (City of Toronto, 2019).

#### Neighbourhood-level marginalisation

Exposure to neighbourhood-level marginalisation was measured by the Ontario Marginalisation Index (ON-Marg). The ON-Marg is a validated index that captures indicators of marginalisation for the following four dimensions: (i) *material deprivation* (area levels of poverty and inability to access and obtain basic material need), (ii) *residential instability* (housing or family instability), (iii) *dependency* (proportion of people who are not receiving income from paid employment or not being compensated for their work), and (iv) *ethnic concentration* (proportion of people who are immigrants and/or identify as belonging to a visible minority group). These dimensional indices were derived from 18 variables from the Canadian census using principal component factor analysis of 42 possible census variables (Matheson et al., 2012). Each neighbourhood was assigned a quintile for each marginalisation dimension, ranging from 1 (lowest level of marginalisation) to 5 (highest level of marginalisation).

#### Neighbourhood-level social capital

Neighbourhood voter participation in a municipal election was used as a proxy indicator of social capital. This indicator was used in previous studies looking at both incidence and health service use in relation to psychosis (Heslin et al., 2018; Kirkbride et al., 2007). The Urban HEART provides a measure of voter participation for each of the 140 neighbourhood for the 2010 municipal election, calculated as percent of eligible voters who voted in the municipal election, using data from the Toronto Election & Registry Services, Toronto Open Data. In Canada, elections are non-compulsory, and in the Toronto municipal elections people vote to elect a mayor and city councillors based on wards (during the period of this study there were 44 wards). There are between 2600 and 5100 eligible voters in each ward, and between 2000 and 2900 eligible voters in each neighbourhood which are smaller areas when compared to the wards (Siemiatycki & Marshall, 2015). All Canadian citizens who are at least 18 years old and are either a resident of Toronto or a property owner can vote in the municipal election. Voter participation ranged between 34.5% and 58.3% across the neighbourhoods, and quintiles were calculated based on participation rates and ranged from the lowest level of social capital in quintile 1 (34.5–41.4%), moderate-low levels of social capital in quintile 2 (41.6–43.9%), moderate levels in quintile 3 (44–47%), moderate-high levels in quintile 4 (47.1–51.5%), and the highest level of social capital in quintile 5 (51.7–58.3%).

### Statistical analysis

Baseline characteristics of the sample were described with descriptive statistics, specifically means and standard deviations (s.d.) for continuous data and proportions for categorical data.

We calculated the crude incidence per 100 000 person-years for the entire city as well as each of the 140 neighbourhoods. These estimates were standardised by age and sex to the 1996 Canadian population to facilitate comparison across neighbourhood by adjusting to the age structure of the standard population. The 1996 census was used as this was the last census prior to cohort entry. To visualise the incidence of psychotic disorders across the city, we first calculated standardised incidence ratios for each neighbourhood based on the population at risk, and proceeded to map the results following smoothing using a Global Empirical Bayes rate estimation approach. This approach smooths each observed neighbourhood rate towards the global city-wide mean to stabilise small area estimates, which can be unstable without smoothing (Pringle, 1996).

Prior to building any regression models, both the mean and variance of the distribution of the outcome were assessed. Both Poisson and negative binomial regression models were constructed and the data were tested for overdispersion. Considering no overdispersion was present, we proceeded to fit Poisson regression models. All further models were fit as mixed Poisson regression models to obtain incidence rate ratios (IRRs) with 95% confidence intervals (CIs), while accounting for neighbourhood-level variance as a random intercept.

Model building proceeded in an iterative manner. We initially fit a null model with no predictors using neighbourhood as a random intercept. A median incidence rate ratio (MIRR) was then calculated based on the null model to provide a descriptive statistic of the median change in the IRR at the neighbourhood level. The MIRR is a measure of a general contextual effect (Austin, Wagner, & Merlo, 2017, 2018) that reflects the influence of the neighbourhood context on the outcomes of interest without specifically assessing the role of covariates. It can be interpreted as the median increase in the IRR as one moves from an area with a low rate of psychotic disorders to a high rate area. A higher MIRR indicates greater variability at the neighbourhood level. The null model was also used as a baseline model to assess the fit of subsequent models.

We then proceeded to fit a model adjusting for age, sex, and all neighbourhood marginalisation indicators, using the lowest quintile as the reference category. Next, a model was fit including all the same variables, with the addition of neighbourhood-level social capital, using the lowest quintile as the reference category. Sex-stratified models adjusting for age and subsequently marginalisation and social capital indicators were also fit to examine effect modification by sex (which has been previously described in the literature). Model fit was assessed using the Akaike information criterion (AIC), where lower AIC values indicate a better model fit.

Stata version 13 was used to fit all regression models. Model estimates are presented as adjusted incidence rate ratios (aIRRs) with 95% CIs. Estimates are considered significant when the CIs do not overlap with unity. Mapping was conducted using R version 3.61 and the spdep package (Bivand & Wong, 2018).

#### Ethics approval

This study obtained ethics approval from the research ethics board at the Center for the Addiction and Mental Health, Toronto, Canada.

## Results

The initial province-wide cohort included 4 284 694 people, of whom 50% were male (*n* = 2 158 166). We excluded 0.7% (*n* = 32 017) of people due to missing postal code information as neighbourhood-level data linkage was not possible. There were 25 686 incident cases of schizophrenia and schizoaffective disorder in the province-wide cohort. The analytic cohort included 649 020 people who lived in one of the 140 Toronto neighbourhoods at the start of the cohort period, of whom 330 475 (51%) were male and 318 545 (49%) were female. The baseline characteristics of the cohort and incident cases are presented in Table 1. Of the 4841 incident cases of schizophrenia and schizoaffective disorder during the follow-up period, 3026 (63%) were males and 1815 (37%) were female. The mean age at diagnosis was 33.0 (s.d. = 8.5 years).

**Table 1.** Sociodemographic characteristics of cohort aged 14–40 years living in Toronto as of 1 April 1999

Mean, ±s.d. and no. (%)						
Characteristic	Toronto population (*n* = 649 020)	Male (*n* = 330 475)	Female (*n* = 318 545)	All incident cases (*n* = 4 841)	Incident male cases (*n* = 3 026)	Incident female cases (*n* = 1 815)
Age at cohort entry (years)	28.6 ± 7.7	28.6 ± 7.7	28.6 ± 7.6	27.4 ± 7.9	26.6 ± 8.0	28.9 ± 7.5
Age at index diagnosis (years)	–	–	–	33.0 ± 8.5	32.1 ± 8.5	34.6 ± 8.1
Marginalisation (quintiles)						
Instability						
1 (lowest)	158 736 (24.5)	80 744 (24.4)	77 992 (24.5)	972 (20.1)	608 (20.1)	365 (20.1)
2	151 981 (23.4)	77 148 (23.3)	74 833 (23.5)	1105 (22.8)	692 (22.9)	413 (22.8)
3	128 523 (19.8)	65 839 (19.9)	62 683 (19.7)	948 (19.6)	590 (19.5)	358 (19.7)
4	121 971 (18.8)	61 838 (18.7)	60 133 (18.9)	1014 (21.0)	618 (20.4)	396 (21.8)
5 (highest)	87 811 (13.5)	44 906 (13.6)	42 904 (13.5)	802 (16.6)	518 (17.1)	284 (15.7)
Deprivation						
1 (lowest)	105 727 (16.3)	51 673 (15.6)	54 054 (17.0)	636 (13.1)	383 (12.7)	253 (14.0)
2	121 130 (18.7)	61 736 (18.7)	59 394 (18.7)	906 (18.7)	567 (18.7)	339 (18.7)
3	151 382 (23.3)	76 986 (23.3)	74 396 (23.4)	1069 (22.1)	676 (22.3)	393 (21.7)
4	149 975 (23.1)	77 247 (23.4)	72 728 (22.8)	1179 (24.35)	738 (24.4)	441 (24.3)
5 (highest)	120 808 (18.6)	62 833 (19.9)	57 973 (18.2)	1051 (21.7)	662 (21.9)	389 (21.4)
Ethnic concentration						
1 (lowest)	120 587 (18.6)	59 011 (17.9)	61 576 (19.3)	755 (15.6)	440 (14.5)	315 (17.4)
2	110 950 (17.1)	57 015 (17.3)	53 935 (16.9)	954 (19.7)	642 (21.2)	312 (17.2)
3	149 593 (23.1)	76 432 (23.1)	73 161 (23.0)	1172 (24.2)	718 (23.7)	454 (25)
4	109 274 (16.8)	56 072 (17)	53 201 (16.7)	800 (16.5)	507 (16.8)	293 (16.1)
5 (highest)	158 618 (24.4)	81 945 (24.8)	76 672 (24.1)	1160 (24.0)	719 (23.8)	441 (24.3)
Dependency						
1 (lowest)	109 682 (16.9)	56 219 (17.0)	53 463 (16.8)	987 (20.4)	642 (21.2)	345 (19.0)
2	131 812 (20.3)	66 852 (20.2)	64 959 (20.4)	1010 (20.9)	620 (20.5)	390 (21.5)
3	127 576 (19.7)	64 999 (19.7)	62 576 (19.6)	951 (19.6)	577 (19.1)	374 (20.6)
4	163 251 (25.2)	83 065 (25.1)	80 186 (25.2)	1067 (22.0)	693 (22.9)	374 (20.6)
5 (highest)	116 701 (18.0)	59 340 (18.0)	57 361 (18.0)	826 (17.1)	494 (16.3)	332 (18.3)
Municipal voting (quintiles)						
1 (lowest)	130 250 (20.1)	67 122 (20.3)	63 128 (19.8)	887 (18.3)	552 (18.2)	335 (18.5)
2	131 000 (20.2)	67 215 (20.3)	63 784 (20.0)	988 (20.4)	610 (20.2)	378 (20.8)
3	130 128 (20.0)	67 472 (20.4)	62 655 (19.7)	1117 (23.1)	737 (24.4)	380 (20.9)
4	130 099 (20.0)	65 070 (19.7)	65 029 (20.4)	926 (19.1)	562 (18.6)	364 (20.1)
5 (highest)	127 545 (19.7)	63 596 (19.2)	63 949 (20.1)	923 (19.1)	565 (18.7)	358 (19.7)

The age-standardised incidence rate of schizophrenia and schizoaffective disorder in Toronto was 69.3 per 100 000 person-years. The age-standardised incidence rate was 52.0 per 100 000 person-years for females, and 86.3 per 100 000 person-years for males. Across the Toronto neighbourhoods, the adjusted incidence rates ranged from 0 per 100 000 person-years to 232.4 per 100 000 person-years. One neighbourhood was an outlier, as it contributed a small amount of person-time years to the cohort with no incident cases. There were three neighbourhoods which had only male incident cases, and two neighbourhoods did not contribute any person-time years to the cohort and had no incident cases.

**Fig. 1.**
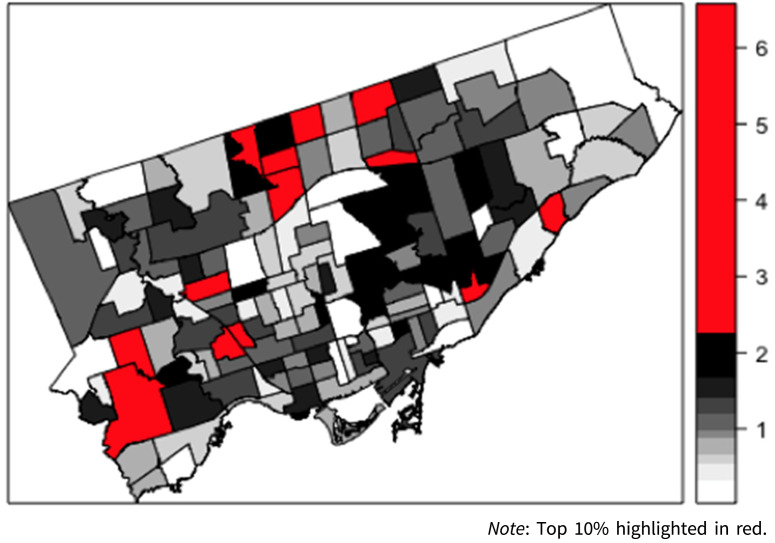
Map of empirical Bayes rate estimates of incident case of schizophrenia and schizoaffective disorder by neighbourhood in Toronto, Canada (1999–2008).

A map of the empirical Bayes rate estimates of the incidence of schizophrenia and schizoaffective disorder by Toronto neighbourhood is presented in Fig. 1, which highlights elevated rates in neighbourhoods outside of the well-resourced downtown area.

The MIRR, which provides an estimate of the magnitude of the contextual effect of neighbourhood, was 1.27. This indicates that we can expect the IRR of schizophrenia and schizoaffective disorder in the neighbourhoods with the highest IRRs to be 27% higher than expected when compared to neighbourhoods with the lowest IRRs.

When examining the impact of marginalisation, all neighbourhood-level marginalisation indicators, other than ethnic concentration, were associated with the risk of developing schizophrenia and schizoaffective disorder (Table 2). People who live in neighbourhoods with high levels of instability (Q2: aIRR = 1.22, 95% CI 1.08–1.40; to Q5: aIRR = 1.49, 95% CI 1.27–1.72) and deprivation (Q2: aIRR = 1.26, 95% CI 1.09–1.46; to Q5: aIRR = 1.55, 95% CI 1.30–1.84), compared to neighbourhoods with the lowest quintiles (Q1), had an increased risk of developing a psychotic disorder. When compared to the lowest quintile of neighbourhood-level dependency (Q1), people living in areas with moderate (Q3: aIRR = 0.86, 95% CI 0.75–0.99) and moderate-high (Q4: aIRR = 0.79, 95% CI 0.69–0.92) levels of dependency had a lower risk of developing a psychotic disorder. Ethnic concentration, across all quintiles (Q2: aIRR = 1.07, 95% CI 0.92–1.25; to Q5: aIRR 1.00, 95% CI 0.85–1.17), was not associated with the risk of developing a psychotic disorder.

**Table 2.** Adjusted incidence rate ratios of schizophrenia and schizoaffective disorder by marginalisation factors and social capital in Toronto, Canada accounting for spatial clustering

	Model 1	Model 2
	aIRR (95% CI)	aIRR (95% CI)
Marginalisation		
Instability (quintiles) 1	Ref.	Ref.
2	**1.22 (1.08–1.40)**	**1.22 (1.08–1.38)**
3	**1.17 (1.02–1.35)**	1.12 (0.97–1.26)
4	**1.29 (1.13–1.48)**	**1.28 (1.11–1.45)**
5	**1.49 (1.27–1.72)**	**1.49 (1.28–1.73)**
Deprivation (quintiles) 1	Ref.	Ref.
2	**1.26 (1.09–1.46)**	**1.27 (1.10–1.46)**
3	**1.27 (1.08–1.48)**	**1.24 (1.06–1.44)**
4	**1.39 (1.18–1.65)**	**1.39 (1.19–1.64)**
5	**1.55 (1.30–1.84)**	**1.56 (1.32–1.85)**
Ethnic concentration (quintiles) 1	Ref.	Ref.
2	1.07 (0.92–1.25)	1.08 (0.93–1.26)
3	1.02 (0.87–1.19)	1.00 (0.85–1.19)
4	0.99 (0.84–1.16)	0.99 (0.83–1.18)
5	1.00 (0.85–1.17)	1.01 (0.85–1.21)
Dependency (quintiles) 1	Ref.	Ref.
2	0.90 (0.79–1.03)	0.91 (0.80–1.04)
3	**0.86 (0.75–0.99)**	**0.86 (0.76–0.99)**
4	**0.79 (0.69–0.92)**	**0.81 (0.70–0.94)**
5	0.89 (0.76–1.03)	0.90 (0.77–1.04)
Social capital (quintiles)		
1	–	Ref.
2	–	**1.17 (1.03–1.33)**
3	–	**1.23 (1.08–1.40)**
4	–	1.06 (0.92–1.23)
5	–	1.14 (0.97–1.34**)**

*Note*: Null mode AIC = 56 935

Model 1 AIC = 56 526

Model 2 AIC = 56 521.

When neighbourhood social capital was added to the model, there was minimal to no change to the aIRRs for each of the marginalisation quintiles (Table 2), with only moderate levels of instability (Q3: aIRR = 1.12, 95% CI 0.97–1.26) becoming non-significant. However, we found an higher risk of developing a psychotic disorder in neighbourhoods in the second (Q2: aIRR = 1.17, 95% CI 1.03–1.33), and third quintile (Q3: aIRR = 1.23, 95% CI 1.08–1.40) of voter turnout, but not the fourth (Q4: aIRR = 1.06, 95% CI 0.92–1.23) and fifth quintile (Q5: aIRR = 1.14, 95% CI 0.97–1.34), when compared to areas with the lowest quintile of voter turnout (Q1).

### Sex-stratified analyses

Among males, we found similar findings with respect to the impact of neighbourhood marginalisation and voter turnout, particularly with neighbourhood-level instability and deprivation (Table 3). In the full model including the proxy indicator of social capital, males residing in neighbourhoods in the second quintiles of ethnic concentration (Q2: aIRR = 1.20, 95% CI 1.01–1.44) had a higher risk of developing a psychotic disorder when compared to neighbourhoods in the lowest quintile (Q1). There was an elevated risk of developing a psychotic disorder in males residing in neighbourhoods in the second (Q2: aIRR = 1.17, 95% CI 1.01–1.35), third (Q3: aIRR = 1.34, 95% CI 1.16–1.56) and fifth quintiles (Q5: aIRR = 1.22, 95% CI 1.02–1.47) of voter turnout, but not the fourth quintile (Q4: aIRR = 1.11, 95% CI 0.94–1.31) when compared to areas with the lowest quintile of voter turnout (Q1).

**Table 3.** Age adjusted incidence rate ratios of schizophrenia and schizoaffective disorder by marginalisation factors and social capital in Toronto for males

	Model 1	Model 2
	aIRR (95% CI)	aIRR (95% CI)
Marginalisation		
Instability (quintiles) 1	Ref.	Ref.
2	**1.24 (1.11–1.40)**	**1.22 (1.05–1.40)**
3	**1.19 (1.06–1.35)**	1.11 (0.95–1.30)
4	**1.26 (1.10–1.42)**	**1.21 (1.04–1.41)**
5	**1.52 (1.38–1.82)**	**1.51 (1.27–1.79)**
Deprivation (quintiles) 1	Ref.	Ref.
2	**1.25 (1.12–1.50)**	**1.26 (1.07–1.49)**
3	**1.28 (1.12–1.50)**	**1.24 (1.04–1.47)**
4	**1.45 (1.26–1.72)**	**1.46 (1.21–1.76)**
5	**1.54 (1.32–1.82)**	**1.58 (1.31–1.91)**
Ethnic concentration (quintiles) 1	Ref.	Ref.
2	**1.18 (1.03–1.36)**	**1.20 (1.01–1.44)**
3	1.02 (0.88–1.20)	1.04 (0.86–1.27)
4	1.03 (0.88–1.20)	1.06 (0.86–1.30)
5	0.99 (0.85–1.16)	1.02(0.83–1.26)
Dependency (quintiles) 1	Ref.	Ref.
2	0.86 (0.74–1.00)	0.88 (0.76–1.02)
3	**0.80 (0.69–0.95)**	**0.82 (0.70–0.95)**
4	**0.79 (0.68–0.94)**	**0.84 (0.71–0.99)**
5	**0.80 (0.68–0.97)**	**0.82 (0.69–0.97)**
Social capital (quintiles)		
1	–	Ref.
2	–	**1.17 (1.01–1.35)**
3	–	**1.34 (1.16–1.56)**
4	–	1.11(0.94–1.31)
5	–	**1.22 (1.02–1.47)**

*Note*: Null mode AIC = 34 368

Model 1 AIC = 34 107

Model 2 AIC = 34 098

Among females (Table 4), results were similar with respect to the impact of neighbourhood-level instability, deprivation, however findings associated with the social capital indicator were unique. Neither neighbourhood-level ethnic concentration nor dependency wasa risk factor forfemales. Only the second quintile of social capital (Q2: aIRR = 1.18, 95% CI 1.00–1.39) was associated with an increased risk in females; however, the associated CI almost straddles unity (1).

**Table 4.** Age adjusted incidence rate ratios of schizophrenia and schizoaffective disorder by marginalisation factors and social capital in Toronto for females

	Model 1	Model 2
	aIRR (95% CI)	aIRR (95% CI)
Marginalisation		
Instability (quintiles) 1	Ref.	Ref.
2	**1.19 (1.01–1.39)**	**1.21 (1.03–1.42)**
3	1.14 (0.97–1.36)	1.15 (0.97–1.37)
4	**1.35 (1.14–1.60)**	**1.35 (1.14–1.60)**
5	**1.49 (1.23–1.80)**	**1.52 (1.26–1.84)**
Deprivation (quintiles) 1	Ref.	Ref.
2	**1.33 (1.10–1.60)**	**1.34 (1.11–1.61)**
3	**1.28 (1.04–1.56)**	**1.28 (1.05–1.57)**
4	**1.38 (1.12–1.72)**	**1.38 (1.11–1.70)**
5	**1.62 (1.30–2.01)**	**1.60 (1.29–1.99)**
Ethnic concentration (quintiles) 1	Ref.	Ref.
2	0.90 (0.74–1.09)	0.87 (0.71–1.07)
3	1.00 (0.82–1.21)	0.95 (0.76–1.17)
4	0.91 (0.75–1.12)	0.87 (0.69–1.09)
5	1.02 (0.83–1.25)	0.98 (0.78–1.24)
Dependency (quintiles) 1	Ref.	Ref.
2	0.95 (0.81–1.12)	0.95 (0.82–1.12)
3	0.94 (0.80–1.12)	0.93 (0.79–1.10)
4	0.77 (0.65–0.92)	0.74 (0.61–0.89)
5	1.00 (0.83–1.21)	1.00 (0.83–1.21)
Social capital (quintiles)		
1	–	Ref.
2	–	**1.18 (1.00–1.39)**
3	–	1.07 (0.90–1.26)
4	–	0.99 (0.82–1.20)
5	–	1.01 (0.83–1.24)

*Note*: Null mode AIC = 22 367

Model 1 AIC = 22 346

Model 2 AIC = 22 348

## Discussion

This study found that the risk of schizophrenia and schizoaffective disorder in Toronto varies by neighbourhood, and is also associated with neighbourhood-level marginalisation and a proxy indicator of social capital. As hypothesised, marginalisation is associated with psychosis risk; however, different forms of marginalisation have a differential impact on risk. From a geographical perspective, areas with the greatest risk were located in areas outside of the downtown core of the city. Although social capital was associated with a higher risk of developing schizophrenia and schizoaffective disorder, the association was not what we initially hypothesised and was not linear in this sample. Moreover, we did not find social capital to substantially attenuate risk associated with marginalisation. These findings provide insight into the role of socioenvironmental risk factors in the development of psychotic disorders and highlight areas that warrant further study.

### Neighbourhood marginalisation

We found different dimensions of marginalisation were associated with the incidence of schizophrenia and schizoaffective disorder in different ways. Higher levels of neighbourhood instability and deprivation were associated with a higher risk of developing a psychotic disorder, yet we did not find ethnic concentration at the neighbourhoodlevel to be associated with the incidence of psychotic disorders in Toronto. Although immigrants and racialised communities are generally known to haveat a higher risk of developing a psychotic disorder, previous research in Ontario has shown that the excess risk may not be the same for all communities and immigrant groups, with some groups even having a lower risk of psychosis (Anderson et al., 2015). It is possible that our finding that ethnic concentration was not associated with an increased risk may be driven by the fact the largest minority groups and a large proportion of the population in some neighbourhood are from East Asian backgrounds (Statistics Canada, 2017), which has been identified as a lower risk group at the population level (Anderson et al., 2015). It is also important to note that we only looked at the association of ethnic concentration across the entire cohort and it may be possible that ethnic concentration may be protective for specific minority communities and groups. Unfortunately, we did not have data on individual-level immigration history, ethnicity, or a specific breakdown of immigrant groups in each neighbourhood. These factors may be important in driving these findings and further examination of the relationship between individual and area-level factors is warranted. Further focus on ethnic density and ethnic concentration with more specific measures is an important area for future study, particularly in the North American context, which may be different to other jurisdictions where this has been studied.

Neighbourhood-level dependency was found to have a protective effect. The findings associated with dependency may, in part, be related to the fact that this marginalisation dimension is based off of multiple census measures (e.g. proportion of the population who are of the age 65+, ratio of the population total population aged 0–14 and 65+ to total population aged 15–64, and proportion of the population aged 15+ not participating in the workforce). It is possible that the individual aspects of the dimension may be associated with risk in different ways and specific indicators may be more important drivers. For example, dimensions based on census measure that have age cut-offs, such as proportion of the population aged 65 and older as well as those younger than 14 years, would be anticipated to be associated with a lower risk of developing schizophrenia and schizoaffective disorder which commonly have an onset in the early adult and late teenage years. Further examination and decoupling of these indicators should be considered in future studies (Zygmunt et al., 2020).

From a geographic perspective, the neighbourhoods that we identified as being at higher risk for psychotic disorders aligns with the general spatial distribution of marginalisation and inequity that exists in Toronto (Hulchanski, 2007). The locations of the neighbourhoods that have the highest risk appear to be outside the catchment areas of the largest specialised mental health services that would benefit this population. Further research to ascertain the relationship between risk and geography (Duncan et al., 2020; McDonald et al., 2020) is important to better plan and deliver evidence-based services that have been shown to improve outcomes in this population (Anderson et al., 2018; Kozloff et al., 2020), and to inform preventative measures (Murray et al., 2020).

### Neighbourhood social capital

Neighbourhood-level social capital was not found to attenuate the impact of marginalisation as we initially hypothesised. Furthermore, we found an increase in the risk of developing schizophrenia and schizoaffective disorder only in neighbourhoods with intermediate levels of voter turnout, relative to those with low voter turnout.

Similar to previous research, we did not find a linear relationship between the risk of developing schizophrenia and schizoaffective disorder and social capital. Previous research has found the risk of developing a psychotic disorder to be decreased in areas with intermediate levels of social capital, suggestive of a ‘U-shaped’ association (Kirkbride et al., 2008), whereas our study found an increased risk in areas with intermediate levels of social capital. However, it is important to note that the association with social capital differed between males and females, and the reversed ‘U-shape’ association was not present when analysis was undertaken in only the females or males. This may be in part due to small numbers in the strata, as well as the use of dissimilar measures and indicators. However, our results were also dissimilar to findings of an earlier studying conducted using a similar voter turnout proxy indicator, which found a linear association between increasing voter turnout and lower risk of psychotic disorder (Kirkbride et al., 2007).

We also found that the impact of social factors, specifically neighbourhood social capital, differs by sex. Our proxy measure of social capital was associated with psychosis risk in males, but not in females. These findings differ from a previous study which found higher rates of first-episode psychosis in females residing in neighbourhoods with lower levels of social capital, and a non-significant trend in the full study sample (O'Donoghue et al., 2016a). However, the proxy measure of social capital in that study was based on area levels of voluntary work rather than voter participation. It is entirely likely that different forms of social capital may impact the risk of developing a psychotic disorder in different ways, considering the differential impact of different forms of social capital on symptoms of psychosis in a non-clinical sample (Freeman et al., 2011). Once the relationship between specific forms of social capital and risk is further clarified, it may be important to study how specific forms of social capital may impact clinical care and service use (Heslin et al., 2018) as well as different forms of recovery (Rotenberg, 2019) in diverse clinical populations.

### Strengths and limitation

Strengths of this study include the use of population-level data and a validated algorithm to identify incident cases of schizophrenia and schizoaffective disorder. The relatively high rate of these disorders (particularly when compared to non-population based cohorts) may be in part due to these methods (Jongsma, Turner, Kirkbride, & Jones, 2019). Furthermore, heterogeneity in rates between different jurisdictions, and even areas within a city, may also be in part due to social and environmental factors impacting rates. From a methodological perspective, we used Bayesian methods to visualise the data that accounts for limitations in mapping small-area level data of rare events. Multilevel modelling was used to appropriately account for area-level variation to obtain adjusted risk estimates. We also used a multi-dimensional measure of neighbourhood-level marginalisation that is based on census data.

Our conclusions are limited by the fact that movement between neighbourhoods and outside of the city, as well as time-varying exposures, are not accounted for. We were also unable to account for other known risk factors, including family history of a mental disorder (Binbay et al., 2012). We used a proxy measure of area-level social capital that focused on only one dimension of social capital. It should also be noted that some population subgroups, particularly new immigrants, may not be eligible to vote (Siemiatycki, 2015) and specific immigrant and ethnic minority communities may have different levels of participation in elections (Milan, 2005). Although theuse of proxy indicators is not uncommon in the literature, future research should focus on measuring social capital directly and measuring multiple dimensions of social capital which may have different association with risk of psychotic disorders. We also would like to note that the data used for the current study are now over 10 years old and warrant replication considering social changes over the course of the past decade.

## Conclusions

The risk of developing schizophrenia and schizoaffective disorder in Toronto, Canada varies by neighbourhood and is associated with neighbourhood-level marginalisation and social capital. The relationship between social capital and risk is not linear, and we found evidence of effect modification by sex. Although this study adds to the current literature on social capital and psychosis from a North American perspective, further research is required to better understand these relationships and consider how this may possibly inform interventions and planning of services. Future research should examine the relationships observed in this study with different dimensions of social capital, and better account for known risk factors, including immigration, ethnicity, and family history of psychosis.
